# Electrical Property Enhancement of a Breast-Fat-Equivalent Phantom for Microwave Mammography

**DOI:** 10.3390/bioengineering13050526

**Published:** 2026-04-30

**Authors:** Kotomi Inada, Yuka Nozaki, Takahiko Yamamoto

**Affiliations:** 1 Department of Electrical Engineering, Graduate School of Science and Technology, Noda Campus, Tokyo University of Science, 2641 Yamazaki, Noda 278-8510, Chiba, Japan; 7325513@ed.tus.ac.jp; 2 Department of Medicinal and Life Sciences, Faculty of Pharmaceutical Sciences, Katsushika Campus, Tokyo University of Science, 6-3-1 Niijuku, Katsushika-ku 125-8585, Tokyo, Japan; nozaki@rs.tus.ac.jp; 3 The Division of Smart Healthcare Engineering, Research Institute for Science and Technology, Tokyo University of Science, 2641 Yamazaki, Noda 278-8510, Chiba, Japan; 4 Department of Electrical Engineering, Faculty of Science and Technology, Noda Campus, Tokyo University of Science, 2641 Yamazaki, Noda 278-8510, Chiba, Japan

**Keywords:** phantom, breast-fat-equivalent phantom, relative permittivity, conductivity, microwave mammography, coaxial probe method

## Abstract

(1) Background: Breast cancer is the most prevalent cancer among women. Conventional screening method have drawbacks, including pain and radiation exposure. Microwave mammography has emerged as a promising diagnostic modality, and its development involves assessing equipment performance; however, ethical concerns limit its use on actual animals or humans. Therefore, an electromagnetic phantom mimicking the relative permittivity and conductivity of the human body has become crucial. (2) Methods: In this study, the electrical properties of a phantom were adjusted by modifying the material composition and additives based on a previous study. We used a network analyzer and dielectric probe to measure the electrical properties using the coaxial probe method. (3) Results: One issue with the existing phantom was the large average error rate in conductivity. Therefore, we increased the conductivity by adding sodium chloride (NaCl). Additionally, we investigated the effects of the amounts of cooking oil, TX-151, and detergent on the electrical properties to ensure a stronger correlation with target values. (4) Conclusions: The average error rates for the relative permittivity and conductivity were 8.26% and 16.9%, respectively, demonstrating an improvement in the agreement with the target values compared to the previous formulations.

## 1. Introduction

The breast is the most common site of cancer in women [1,2,3]. A conventional screening method for breast cancer is X-ray mammography [4]. However, such an examination method involves problems such as pain during examination and radiation exposure [5]. Another significant challenge of conventional screening is the limited diagnostic accuracy for dense breasts [6,7,8]. Dense breast, which is prevalent among Asian women, refers to a condition with a high percentage of mammary glands. Consequently, it is difficult for clinicians to accurately detect lesions using X-ray mammography, as both cancerous tissue and mammary glands appear white on the images.

Recently, microwave mammography [9,10,11] has attracted attention as a new examination method. It is a cancer detection method based on the difference in dielectric constants between cancerous and normal tissues. In contrast to conventional methods, this approach does not cause pain or exposure during the examination and is said to have high diagnostic accuracy for highly concentrated breasts.

Evaluating functional performance is critical in the development of medical devices. However, research methodologies that use real animals or human subjects are often impractical for ethical reasons. Medical phantoms can be useful alternatives to living tissue in such applications [12]. Several types of medical phantoms have been developed, such as radiation [13], ultrasound [14,15] and electromagnetic phantoms. In this study, we consider an electromagnetic phantom that simulates the relative permittivity, $\varepsilon_{r}^{\prime }$, and conductivity, σ, of the human body (referred to here as “electrical properties”) [16]. Electromagnetic phantoms have been developed in liquid [17], solid [18,19], and semi-solid [20] forms. In particular, semi-solid gel phantoms with high water content feature easy prototyping, a high degree of freedom with regard to shape, and low cost. To take advantage of these characteristics and develop prototypes that are easy to fabricate and cost-effective, this study focused on developing high-hydrous gel phantoms.

To overcome the aforementioned limitations of conventional methods, developing a comprehensive breast cancer model phantom—integrating skin, mammary glands, breast fat, and tumor tissues—is essential for evaluating new modalities like microwave mammography. As a fundamental step toward this goal, this study focuses on improving the electrical property accuracy of a breast-fat-equivalent phantom, which represents a major portion of the breast volume.

Based on our previous work [21,22], which originally focused on general fat-equivalent phantom [23], this study aimed to develop a refined breast-fat-equivalent phantom. The novelty of this research lies in achieving property matching across a broad frequency range of 1–15 GHz [8], covering the entire microwave band. Furthermore, this study provides new insights by clarifying the short-term temporal changes in electrical properties—a factor that has remained unclear in previous research—thereby defining the appropriate operational period for reliable measurements.

The core development concept focuses on extreme ease of fabrication: the phantom is exclusively composed of readily available and safe materials, such as purified water, cooking oil, and detergent. Furthermore, only a simple and accessible preparation procedure is required, with no specialized laboratory equipment or complex chemical processing. This emphasis on simplicity and ease of prototyping ensures that the proposed phantom serves as a highly accessible tool for the rapid, preliminary evaluation of microwave mammography systems.

## 2. Materials and Methods

### 2.1. Measurement of Electrical Properties

We used a network analyzer (N5230A, Agilent Technologies, Santa Clara, CA, USA) and a dielectric probe (Agilent Technologies, 85070D) to measure electrical properties using the coaxial probe method [24,25,26]. This method measures the complex relative permittivity of the sample by measuring the reflection coefficient at the sample interface, allowing for measurements over a wider bandwidth than other measurement systems (such as the parallel-plate method [27,28] or coaxial-tube transmission method [29]), and the measurement system is relatively simple. The coaxial probe method is also suitable for measuring liquid and semi-solid materials because the sample is held in close contact with the probe endpoints.

**Figure 1:**
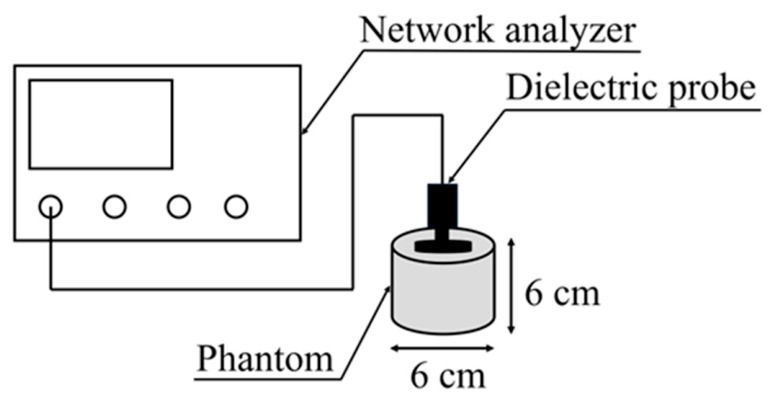
Schematic of the measurement system consisting of a network analyzer, dielectric probe, and phantom (6 cm in diameter and 6 cm in height).

Figure 1 shows a schematic of the measurement system. The measurement system was calibrated using the following three steps: (1) open in air, (2) shortening with a calibration block, and (3) immersion in purified water at 25 °C. Measurements were conducted with a room temperature of 24 °C and humidity of 39%. The measurement frequency ranged from 1 GHz to 15 GHz, with a phantom temperature of 22 °C [16] during measurement. The dimensions and shape of the phantom were adopted as a cylinder with a diameter of 6 cm and height of 6 cm, determined based on the specifications for dielectric probes [30].

The complex relative permittivity, $\varepsilon_{r}^{*}$, is measured using a network analyzer and dielectric probe. From the measured complex relative permittivity, the relative permittivity (real part), $\varepsilon_{r}^{\prime }$, and conductivity, σ, are derived using Equations (1) and (2):

(1) $$\varepsilon_{r}^{*}=\varepsilon_{r}^{\prime }-j\varepsilon_{r}^{\prime \prime },$$

(2) $$\sigma =\omega \varepsilon_{0}\varepsilon_{r}^{\prime \prime }=2\pi f\varepsilon_{0}\varepsilon_{r}^{\prime \prime }\left[S/m\right],$$

where $\varepsilon_{r}^{\prime }$ represents the relative permittivity; $\varepsilon_{r}^{\prime \prime }$, the dielectric loss (imaginary part); $\omega \left[rad/s\right]$, the angular frequency; f[Hz], the measurement frequency; and $\varepsilon_{0}\;[F/m]$, the permittivity of the vacuum (=$8.854\times 10^{-12}\;F/m$). These parameters, $\varepsilon_{r}^{\prime }$ and σ, are the primary electrical properties used for tumor detection in microwave mammography.

Furthermore, the error rate relative to the target value during the development process was calculated using Equation (3).

(3) $$\begin{array}{c} \left(Error\;rate\right)=\frac{\left|\left(Measured\;value\right)-\left(Target\;value\right)\right|}{\left(Target\;value\right)}\times 100\;\left[\%\right] \end{array}$$

Specifically, the average error rate was defined as the mean of the error rates across the 1–15 GHz range. Naturally, an average error rate closer to zero indicates better agreement with the target values.

### 2.2. Materials and Methodology for Phantom Fabrication

We prototyped the phantom by blending multiple materials. The materials used in this study and the effects of each material are listed in Table 1.

The methodology for fabricating the phantom is as follows.

1. Sodium chloride (NaCl) was gradually added to a vessel filled with purified water and thoroughly mixed.
2. In order, agar, detergent, and cooking oil were gradually added to a vessel and thoroughly mixed. The solution was then rapidly heated once to a high temperature while stirring.
3. Heating was terminated as soon as the solution started boiling. TX-151 was added, and the mixture was thoroughly mixed.
4. The phantom was poured into a container and then allowed to cool and solidify.

The mixture was manually stirred using a whisk, with careful attention paid to the bottom of the vessel during heating to prevent scorching. Heating was performed at approximately 780 W for 3–5 min until the solution reached its boiling point. After heating, the phantom was placed in a refrigerator (IRSN-32B-S, IRIS OHYAMA) for 1–1.5 h to solidify. Prior to measurement, the phantom was allowed to stabilize at room temperature for 1–2 h until it reached a uniform temperature of 22 °C. The total fabrication time, excluding cooling and stabilization, was approximately 12 min.

## 3. Results

### 3.1. Current Status of Fat-Equivalent Phantom

In this study, we attempted to develop a breast-fat-equivalent phantom by refining a phantom originally designed to mimic the characteristics of general fat tissue in our previous research [21]. The composition of the general fat-equivalent phantom proposed in the previous study [21] is summarized in Table 2.

One of the materials listed is detergent. While a neutral dishwashing detergent was used in the previous study [21], a subsequent investigation [22] revealed that a weakly acidic detergent is more suitable for improving shape retention.

**Figure 2:**
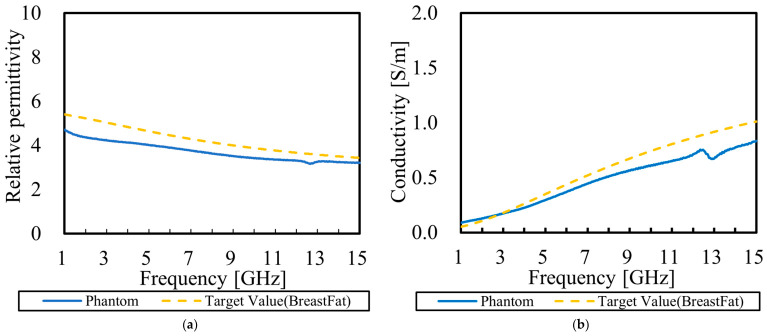
Electrical properties of the prototype phantom compared with the target values of breast fat: (**a**) measured relative permittivity as a function of frequency; (**b**) measured conductivity as a function of frequency.

Based on these findings, we first fabricated a prototype phantom by maintaining the addition amounts proposed in the previous study [21] but changing the type of detergent from neutral to weakly acidic. The electrical properties of this prototype were measured and compared with the target values for breast fat, which is a specific focus of this study. The measurement results are shown in Figure 2. The target values were determined based on the ‘Breast Fat’ data obtained from Ref. [23].

The measurement results show that the average error rates against the target values are 12.1% for relative permittivity and 18.0% for conductivity.

### 3.2. Development of Breast-Fat-Equivalent Phantom

Based on the measurement results presented in Section 3.1, we attempted to improve the electrical properties by adjusting the addition amount of each constituent material. In particular, given the critical limitation of previous phantoms being unable to achieve sufficient conductivity, this study focused on increasing the conductivity to more closely align with the target values.

Furthermore, as the shelf life during which the electrical properties remain stable was not clarified for previous phantoms, this study also investigated the stable operational period of the developed phantom.

#### 3.2.1. Effect of NaCl Addition on Conductivity

**Figure 3:**
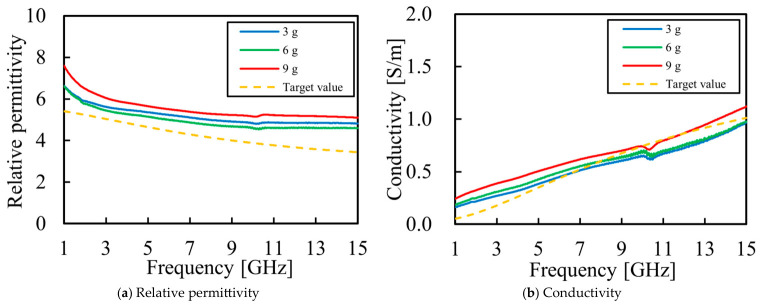
Electrical properties of the phantom compared with the target breast fat values, with the amount of NaCl varying from 3 g to 9 g: (**a**) measured relative permittivity as a function of frequency; (**b**) measured conductivity as a function of frequency.

We attempted to increase conductivity by adding NaCl, which was found to be effective in increasing conductivity in a previous study [31]. The compositions considered are listed in Table 3, and the measurement results are shown in Figure 3.

#### 3.2.2. Effect of Cooking Oil Amount on Electrical Properties

From the results in Section 3.2.1, it is clear that the increase in relative permittivity due to the addition of NaCl must be suppressed. Based on the measurement results in Section 3.2.1, the amount of NaCl added was 6 g, which had the minimal average error rate. The amount of cooking oil varied from 110 g to 120 g and 130 g, and the electrical properties were measured. The amounts of materials other than cooking oil were equivalent to those listed in Table 3 (b).

The measured results showed that the relative permittivity was successfully reduced by increasing the amount of cooking oil added. The best agreement with the target value was obtained when 130 g of cooking oil was added. The average error rates were 6.29% for permittivity and 35.5% for conductivity. However, the rate of change in conductivity with increasing frequency was small, and the error rate was substantial.

#### 3.2.3. Effect of Amounts of TX-151 and Detergent Added on Electrical Properties

**Figure 4:**
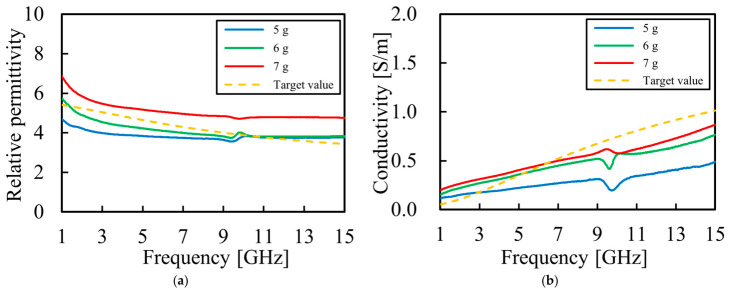
Electrical properties of the phantom compared with the target values of breast fat, with the amount of TX-151 varying from 5 g to 7 g: (**a**) measured relative permittivity as a function of frequency; (**b**) measured conductivity as a function of frequency.

First, the effect of the amount of TX-151 added was investigated. The compositions considered are listed in Table 4, and the measurement results are shown in Figure 4.

The measurement results show that the composition in Table 4 (b) had the best agreement with the target values. The average error rates were 6.29% for relative permittivity and 35.5% for conductivity.

**Figure 5:**
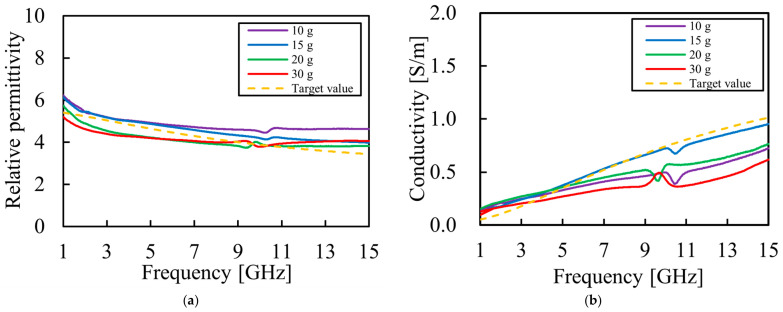
Electrical properties of the phantom compared with the target values of breast fat, with the amount of detergent varying from 10 g to 30 g: (**a**) measured relative permittivity as a function of frequency; (**b**) measured conductivity as a function of frequency.

Next, the amount of detergent added was adjusted based on the TX-151 measurements; 6 g of TX-151 was considered to be the most appropriate amount to add based on the data presented in Figure 4. Therefore, the amounts of materials other than detergent were equivalent to those listed in Table 4 (b). The compositions considered are listed in Table 5, and the measurement results are shown in Figure 5. The detergent used in this study was a weakly acidic detergent obtained from a previous study [22].

The findings of this study indicate that the composition depicted in Table 5 (b) exhibits the strongest concordance with the target value, with average error rates of 8.26% and 16.9% for relative permittivity and conductivity, respectively.

#### 3.2.4. Proposed Breast-Fat-Equivalent Phantom

**Figure 6:**
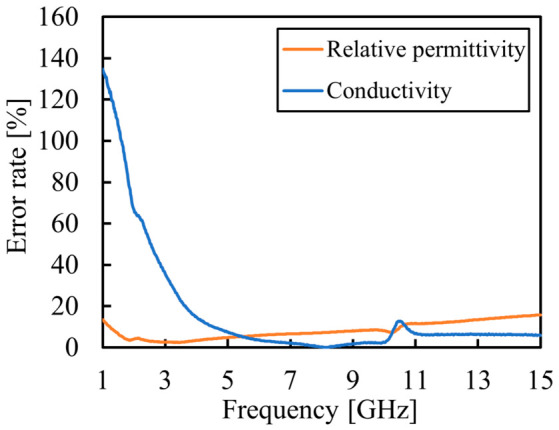
Frequency dependence of the error rates for the relative permittivity and conductivity of the proposed phantom.

The composition of the proposed phantom is listed in Table 6, and the average error rates relative to the target values are presented in Table 7. Furthermore, the frequency dependence of the error rates is shown in Figure 6. As indicated in Table 7, the average error rates of the proposed phantom are lower than those of the phantom before improvement, achieving values of 8.26% for relative permittivity and 16.9% for conductivity. These results represent improved agreement with the target values across the frequency range investigated.

Figure 6 shows that the error rate for conductivity is significantly higher at frequencies below 5 GHz. However, when evaluating the error rates specifically within the 5–20 GHz range, the average error rates were found to be 9.82% for relative permittivity and 4.58% for conductivity. Within this same band, the maximum error rates were 15.9% and 12.7%, respectively. These results demonstrate that the proposed phantom is particularly effective and reliable for applications in the 5–20 GHz range, offering high precision within this high-frequency band.

**Figure 7:**
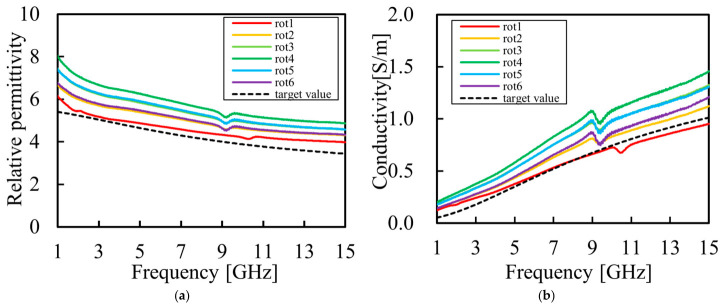
Electrical properties for six independently fabricated phantom specimens to verify reproducibility: (**a**) measured relative permittivity as a function of frequency; (**b**) measured conductivity as a function of frequency.

**Figure 8:**
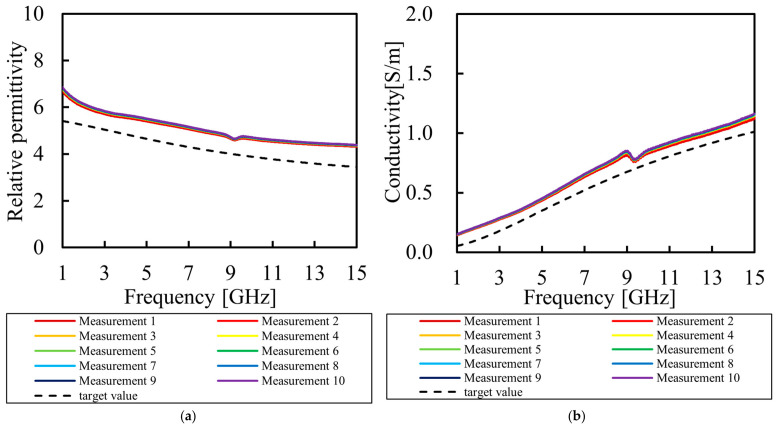
Electrical properties from 10 repeated evaluations on a single phantom (rot 2) specimen to verify intra-specimen homogeneity: (**a**) measured relative permittivity as a function of frequency; (**b**) measured conductivity as a function of frequency.

To verify the reproducibility of the proposed composition, six independent specimens were fabricated using the optimal formulation, and their electrical properties were measured. The results are presented in Figure 7. Furthermore, to confirm the intra-specimen uniformity and the stability of the measurements, repeated evaluations were conducted on a single specimen (“rot 2” from Figure 7). These results are shown in Figure 8.

#### 3.2.5. Temporal Changes in the Electrical Properties of the Proposed Phantom

**Figure 9:**
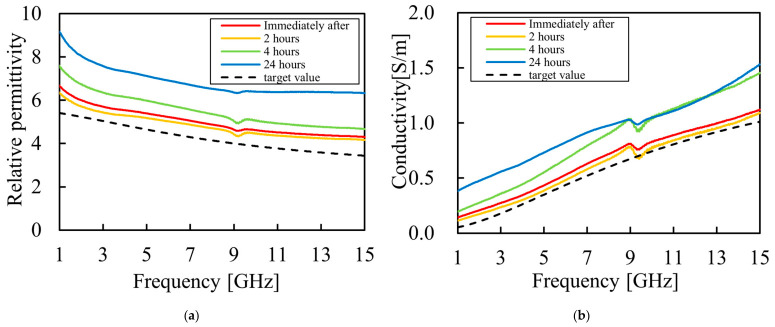
Electrical properties for the phantom (rot 2) to evaluate short-term stability over 24 h: (**a**) measured relative permittivity as a function of frequency; (**b**) measured conductivity as a function of frequency.

To verify short-term stability and determine the appropriate operational period for the proposed phantom, we investigated the changes in electrical properties over a 24 h period following fabrication. Using the same specimen (“rot 2”) evaluated in Section 3.2.4, measurements were conducted immediately after fabrication, as well as at 2, 4, and 24 h post-fabrication. The measurement results are presented in Figure 9.

The measurement results reveal that both relative permittivity and conductivity tend to increase over time. The average error rates for each elapsed time are presented in Table 8. As indicated in Table 8, the electrical properties remained highly stable with no changes for up to approximately two hours post-fabrication. These results demonstrate that the proposed phantom provides reliable performance as an evaluation tool within this two-hour operational window.

## 4. Discussion

### 4.1. Effect of Composition Changes in Each Material on Electrical Properties

#### 4.1.1. Conductivity Control Through the Addition of NaCl

The addition of NaCl effectively increased conductivity. The measurement results (Figure 3) confirmed that, while conductivity increased with higher NaCl concentrations, there was a trade-off where the relative permittivity also increased simultaneously.

#### 4.1.2. Control of Relative Permittivity Using Cooking Oil

The excessively elevated relative permittivity caused by NaCl addition was reduced to an average error rate of 6.29% relative to the target value by increasing the cooking oil content from 110 g to 130 g. Among the materials used in this study, increasing the volume fraction of oil—which has an extremely low relative permittivity—is considered the dominant factor in lowering the effective permittivity of the entire phantom.

#### 4.1.3. Stabilization of Frequency Characteristics Using TX-151 and Detergent

The relative permittivity approached the target value upon adjusting the additive amounts in Section 3.2.1 and Section 3.2.2. However, it became evident that the rate of change in conductivity with increasing frequency was less than the target value. Section 4.1.1 and Section 4.1.2 show that NaCl and cooking oil influenced the relative permittivity. Given the significant value of relative permittivity exhibited by purified water, we hypothesized that substantial fluctuations in relative permittivity may occur if the amount of purified water added was modulated. Therefore, based on the hypothesis that the rate of change in conductivity depends on the amount of TX-151 and detergent added, each additive’s concentration was adjusted. While the efficacy shown in Table 1 indicates that TX-151 primarily functions as a thickening agent with no significant contribution to electrical properties, the measurement results (Figure 4) confirm its influence on electrical characteristics. Specifically, an increase in the amount of TX-151 added resulted in a tendency for both relative permittivity and conductivity to increase. Furthermore, detergent has a surfactant effect, as shown in Table 1. Moreover, the measurement results (Figure 5) confirm its influence on electrical properties. Specifically, an increase in the amount of detergent added was found to cause a tendency for both the relative permittivity and conductivity to decrease. Furthermore, as the additive amount increased, a larger positive slope was observed in the rate of change in conductivity with increasing frequency. The specific mechanism (such as chemical evidence) behind why the detergent additive amount improved the rate of change in conductivity remains unclear and needs to be elucidated in future studies.

### 4.2. The Potential Practicality of the Developed Phantom

The phantom proposed in this study exhibited improved agreement with the target values within the frequency range relevant to microwave mammography. Notably, we succeeded in increasing conductivity—a major challenge in previous formulations—thereby reducing the average error rate by approximately 1% compared to the phantom before improvement. Furthermore, a reduction of approximately 4% in the average error rate was achieved for relative permittivity.

Although further investigations regarding long-term stability, temperature dependence, and durability are necessary, the simple composition using readily available materials suggests that this phantom has the potential to serve as a practical preliminary evaluation tool. Specifically, because it can be fabricated without specialized equipment using inexpensive materials such as purified water, cooking oil, and detergent, it offers a cost-effective solution for initial laboratory testing.

### 4.3. Future Considerations

While this study established a composition for replicating the electrical properties of breast fat tissue, several challenges remain for practical application. First, it is essential to evaluate the temporal changes and long-term stability of the phantom [32]. Because the proposed formulation contains purified water, cooking oil, and surfactants, issues such as dehydration due to water evaporation or phase separation may occur over time. Defining optimal storage conditions—such as the use of airtight containers and precise temperature control—and identifying the characteristic shelf life will be key to enhancing its reliability as a standardized evaluation tool. Along with this, a comprehensive evaluation of durability under repeated measurements is required to ensure structural integrity.

Second, it is necessary to investigate the influence of agar concentration on the electrical properties. In this study, the agar concentration was not treated as a variable, as its primary role was considered to be shape retention. However, our findings suggest that even additives like TX-151 and detergent can influence electrical properties through complex interactions. Therefore, elucidating the underlying physicochemical mechanisms of these interactions is indispensable for achieving more precise control over the phantom’s characteristics. Finally, establishing an appropriate range for the acceptable error rate based on specific clinical requirements will be crucial for validating the phantom as a reliable standard for microwave mammography.

## 5. Conclusions

In this study, we developed a broadband phantom with electrical properties equivalent to those of breast fat, building upon the composition of an general fat-tissue phantom from previous research. A primary challenge with the existing formulation was its inability to achieve sufficient conductivity, resulting in a high average error rate. To address this, we successfully matched the dielectric properties over an exceptionally wide frequency range of 1–15 GHz by increasing the conductivity with NaCl and optimizing the proportions of cooking oil, TX-151, and detergent. The refined formulation achieved average error rates of 8.26% for relative permittivity and 16.9% for conductivity, demonstrating improved agreement with target values compared to previous models.

Furthermore, we clarified the short-term stability of the phantom, identifying a reliable “operational window” of approximately two hours post-fabrication. This provides essential guidelines for ensuring measurement reliability during experimental procedures.

Despite these advancements, several challenges remain for broader application. Future research should focus on evaluating long-term stability and identifying optimal storage conditions to extend the shelf life. Furthermore, it is essential to elucidate the underlying physicochemical mechanisms of how additives like TX-151 and detergent influence electrical properties. Additionally, we intend to investigate the influence of agar concentration, which was previously considered only for shape retention, to further enhance the precision and reproducibility of the phantom.

## Figures and Tables

**Table 1:** Materials and Effects.

Material	Effect
Purified water	Main material
Cooking oil	Decreases relative permittivity
Detergent	Surfactant
Agar	Shape retention
TX-151	Thickening
Sodium chloride (NaCl)	Increases conductivity

**Table 2:** Composition of the fat-equivalent phantom developed in the previous study [21].

Material	Addition Amount [g]
Purified water	60
Cooking oil	110
Detergent	20
Agar	9.0
TX-151	6.0

**Table 3:** Composition of the phantom, with the amount of added NaCl varying from 3 g to 9 g.

Material	Addition Amount [g]		
	(a)	(b)	(c)
Purified water	60	60	60
Cooking oil	110	110	110
Detergent	20	20	20
Agar	9.0	9.0	9.0
TX-151	6.0	6.0	6.0
NaCl	3.0	6.0	9.0

**Table 4:** Composition of the phantom, with the amount of TX-151 addition varying from 5.0 g to 7.0 g.

Material	Addition Amount [g]		
	(a)	(b)	(c)
Purified water	60	60	60
Cooking oil	130	130	130
Detergent	20	20	20
Agar	9.0	9.0	9.0
TX-151	5.0	6.0	7.0
NaCl	6.0	6.0	6.0

**Table 5:** Composition of the phantom, with the detergent addition amount varying from 10 g to 30 g.

Material	Addition Amount [g]			
	(a)	(b)	(c)	(d)
Purified water	60	60	60	60
Cooking oil	130	130	130	130
Detergent	10	15	20	30
Agar	9.0	9.0	9.0	9.0
TX-151	6.0	6.0	6.0	6.0
NaCl	6.0	6.0	6.0	6.0

**Table 6:** Composition of the proposed phantom.

Material	Addition Amount [g]
Purified water	60
Cooking oil	130
Detergent	15
Agar	9.0
TX-151	6.0
NaCl	6.0

**Table 7:** Comparison of average error rates in relative permittivity and conductivity between the phantom before improvement and the proposed phantom.

	Relative Permittivity	Conductivity [S/m]
Proposed phantom	8.26%	16.9%
Before improvement	12.1%	18.0%

**Table 8:** Average error rates of electrical properties for the phantom at each elapsed time post-fabrication.

	Relative Permittivity	Conductivity [S/m]
Immediately after	8.26%	26.7%
2 h	13.8%	17.3%
4 h	30.2%	66.8%
24 h	62.7%	114%

## Data Availability

The authors confirm that all relevant data are included in this article.
